# A Longitudinal Study on the Impact of Preceptors’ Perceived Difficulty and Role Performance in Instructing Newly Graduated Nurses—Following Changes in Clinical Practicum Due to COVID-19—On Their Mental Health

**DOI:** 10.3390/healthcare13192401

**Published:** 2025-09-24

**Authors:** Takashi Ohue, Yuka Ohue

**Affiliations:** 1 Graduate School of Health Sciences and Faculty of Health Sciences, Okayama University, 2-5-1 Shikatacho, Kita-ku 700-8558, Okayama, Japan; 2 Department of Nursing, Faculty of Nursing, Hyogo University, 2301 Shinzaike, Hiraoka-cho, Kakogawa 675-0195, Hyogo Prefecture, Japan; morisaki@hyogo-dai.ac.jp

**Keywords:** COVID-19, preceptor, mental health, longitudinal study

## Abstract

**Highlights:**

**What are the main findings?**

- High perceived instructional difficulty and high role performance were associated with higher stressors, emotional exhaustion, and turnover intentions among preceptors.
- Interaction effects over time revealed that preceptors with both high difficulty and high role performance showed the greatest psychological burden by the end of the study.

**What is the implication of the main finding?**

- Mental health support programs should focus on preceptors with high perceived difficulty and high performance to prevent burnout and turnover.
- Early identification and intervention could help maintain preceptor well-being and workforce stability.

**Abstract:**

Objective: This longitudinal study examined how preceptors’ perceived difficulty and role performance in instructing newly graduated nurses impacted by restricted clinical practicum opportunities because of COVID-19 impact their mental health outcomes, including stressors, burnout, and turnover intention. Methods: The study surveyed 426 preceptors responsible for newly graduated nurses across 39 hospitals during fiscal year 2022. Data were collected at three time points: June, September, and December 2022. The questionnaire assessed personal attributes, perceived instructional difficulty (PID) due to limited clinical practice, self-rated preceptor role performance, nursing job stressors, burnout, and intention to resign. Two-way ANOVA was conducted to analyze the effects of perceived difficulty (high/low) and role performance (high/low) on mental health indicators. Results: Seventy-six preceptors (6 males, 70 females) completed all three surveys. In June 2022, preceptors reporting high perceived difficulty demonstrated significantly higher scores in role performance subscales, including “goal achievement and accident prevention” and “continuation of instruction with cooperation.” Significant main effects of perceived difficulty and role performance were observed on stressors such as role conflict, physician conflict, and death-related stress, as well as on burnout dimensions such as emotional exhaustion and personal accomplishment. By December, significant interaction effects emerged for outcomes related to “intention to quit nursing” and “desire to change departments.” Conclusions: Preceptors’ PID and role performance significantly influence their stress, burnout, and turnover intentions. Those experiencing both high difficulty and high role performance experience increased psychological burdens. This underscores the importance of targeted mental health support for preceptors.

## 1. Introduction

In December 2019, the first case of pneumonia caused by a novel coronavirus disease (COVID-19) was reported in Wuhan, Hubei Province, China. The infection rapidly spread, and in January 2020, the World Health Organization (WHO) declared it a Public Health Emergency of International Concern (PHEIC) [1]. This unprecedented pandemic had a profound impact not only on healthcare systems but also on nursing education.

Clinical practicum is a core component of nursing education and relies on collaboration with hospitals and community medical institutions. It was significantly restricted or canceled because of infection control measures. Consequently, many nursing schools in Japan were forced to revise their curricula, including shortening clinical practice or replacing it with simulation training [2,3].

Clinical practicum is essential for integrating knowledge, skills, and attitudes acquired in foundational and specialized nursing courses into real-world application [4]. The inability to sufficiently participate in clinical training may hinder the development of clinical competency and judgment in newly graduated nurses.

Kwon et al. stated that this study explored nursing students’ clinical practice during the COVID-19 pandemic. Despite fears and restrictions, students gained valuable experience, felt protected, and developed a strong sense of duty as future nurses [5]. Conversely, Palese et al. [6] did not identify a significant difference in basic nursing skills or role performance between newly graduated nurses who experienced restricted training and those from the previous year, indicating variability in outcomes.

Ohue et al. [7] suggested that decreased clinical practicum and increased reliance on in-school training may lead to diminished practical ability among new nurses. This view underscores the importance of both the quality and quantity of hands-on experience.

In this context, newly graduated nurses in 2022 entered clinical settings with limited real-world experience, potentially experiencing difficulties in skill development and workplace adaptation. Consequently, preceptors and nurse educators faced increased instructional burdens, requiring greater personalized—and flexible—guidance.

Indeed, studies have shown that nurses who graduated during the COVID-19 pandemic experienced anxiety about patient care and lacked confidence in clinical decision making, emphasizing the need for support systems [8]. However, the impact of these abrupt changes in educational environments on the mental health and role performance of the preceptors responsible for guiding these nurses remains vague.

Newly graduated nurses who completed their education during the COVID-19 pandemic have been shown to experience anxiety regarding patient care and clinical decision making, as well as difficulties in developing practical skills and adapting to the workplace [7]. Under these circumstances, preceptors and nurse educators face increased instructional burdens, which may elevate the risk of psychological stress and burnout.

Cognitive behavioral therapy (CBT) provides a useful framework for understanding and addressing these stressors and anxieties. CBT posits that an individual’s cognitions (thoughts) influence emotions and behaviors and that modifying distorted cognitive patterns that contribute to stress and anxiety can reduce psychological burdens and promote adaptive behaviors [9,10]. In nurse mental health models, “stressors”, “burnout”, and the “intention to resign” are identified as critical factors. Analyzing these factors from a CBT perspective can provide a foundation for developing strategies to support the psychological well-being of both preceptors and newly graduated nurses.

Mental health models for nurses have identified “stressors,” “burnout,” and “intention to resign” as critical indicators [9]. Clarifying the impact of these factors may induce the development of effective cognitive–behavioral strategies to mitigate stress [10].

While previous studies have mainly focused on the difficulties experienced by newly graduated nurses, limited attention has been paid to the mental health of the preceptors who guide them. Moreover, existing studies on preceptors have primarily addressed instructional challenges in cross-sectional contexts, leaving a gap in understanding how these challenges influence preceptors’ mental health over time. This study uniquely contributes to the literature by addressing this gap through a longitudinal examination of preceptors’ PID, role performance, and mental health during the COVID-19 pandemic.

### Research Objective

Newly graduated nurses in fiscal year 2022 experienced significant changes in their clinical practicum due to COVID-19, including cancellations and a shift to online formats. This study aims to longitudinally examine the impact of preceptors’ role performance and perceived difficulty in instructing these nurses on the preceptors’ mental health.

## 2. Materials and Methods

### 2.1. Participants

The study targeted 426 preceptors involved in guiding newly graduated nurses in the fiscal year 2022 across 39 hospitals in the Hyogo Prefecture, Japan. All hospitals agreed to participate.

### 2.2. Study Design

This study employed a longitudinal research design, conducting surveys at three time points: June, September, and December 2022.

### 2.3. Sampling Method and Sample Size

The study participants were hospital nurses who served as preceptors for newly graduated nurses in 2022 in 39 hospitals in Hyogo Prefecture, Japan. All the participants provided informed consent to participate in the study. Initially, facility administrators were contacted and eligible nurses were recruited through purposive sampling. The required sample size was calculated using the G*Power 3.1 software. Based on the assumptions of a medium effect size (f = 0.25), α = 0.05, and statistical power (1 – β) = 0.80, the minimum sample size was determined to be 52. The target sample size was increased by 30% to account for potential nonresponses or incomplete data, resulting in a minimum requirement of 68 participants. Ultimately, 76 nurses completed all three survey rounds (June, September, and December), comprising six men (7.9%) and 70 women (92.1%) and yielding a final response rate of 17.8%.

### 2.4. Prevention of Information Contamination

Explanatory materials and consent forms were individually distributed to reduce the risk of information contamination. Participants completed the questionnaires independently, and their responses were anonymized to avoid influence from colleagues or supervisors. Furthermore, all data were securely coded and stored to ensure confidentiality and maintain unbiased reporting.

### 2.5. Survey Period

First survey: 1–30 June 2022.

Second survey: 1–30 September 2022.

Third survey: 1–31 December 2022.

Three survey points were chosen to align with the key stages of the first year for newly graduated nurses. In Japan, nurses typically begin employment in April. By June, after the initial orientation and training, they start performing independent tasks, which places greater instructional demands on preceptors. September represents the midpoint of the preceptorship period when new nurses are more accustomed to clinical duties, but preceptors may face ongoing challenges in balancing the guidance of new nurses with their own workload. By December, new nurses are expected to achieve greater autonomy and the intensity of preceptor support often decreases, which may alter preceptors’ perceived difficulty and associated stress.

### 2.6. Survey Contents

#### 2.6.1. Demographic Data

Participants provided demographic data including sex, age, department, academic background, qualifications, and employment type.

#### 2.6.2. PID

Preceptors rated the degree of difficulty experienced in instructing newly graduated nurses who had insufficient clinical practicum because of COVID-19. Responses were recorded on a 5-point Likert scale ranging from “Not at all difficult” to “Very difficult.”

#### 2.6.3. Role Performance as a Preceptor

The “Preceptor Role Self-Evaluation Scale,” developed by Yoshitomi and Funashima [11], was applied to assess the role performance of nurses instructing new graduates. This 35-item scale comprises seven subscales designed to comprehensively evaluate the multifaceted functions of a preceptor:

- Collecting diverse information about the new nurse and developing individualized instructional plans.
- Providing instruction and evaluation based on the instructional plan.
- Alleviating the anxiety of the new nurse and compensating for deficiencies.
- Offering explanations and psychological support to help the new nurse continue working.
- Achieving instructional goals and preventing incidents while monitoring the new nurse’s condition.
- Continuing instruction with support from other staff and patients.
- Managing other duties in addition to preceptorship.

Each item is rated on a 4-point Likert scale from 1 (“Not applicable at all”) to 4 (“Highly applicable”). Higher scores indicate greater self-evaluated role performance as a preceptor. The reliability and validity of the scale have been confirmed.

#### 2.6.4. Mental Health Factors

Job Stressors:

The Nursing Job Stressor Scale (NJSS), developed by Higashiguchi [12], was applied to assess stressors related to nursing practice. It comprises 33 items across seven subscales:

- Conflict with other nurses.
- Role conflict.
- Conflict with physicians/autonomy.
- Dealing with death and dying.
- Qualitative workload.
- Quantitative workload.
- Conflict with patients.
- Higher scores indicate greater perceived stress.

Burnout:

Burnout was assessed using the Maslach Burnout Inventory (MBI), originally developed by Maslach et al. [13] and modified by Tao et al. and Kubo [14]. This scale evaluates three dimensions of burnout: emotional exhaustion, depersonalization, and personal accomplishment. It comprises 17 items, each rated on a 5-point Likert scale ranging from “Always” to “Never.” High scores in emotional exhaustion and depersonalization, combined with low scores in personal accomplishment, indicate a higher likelihood of burnout.

Intention to Resign:

Intention to resign was measured using items based on the categories defined by Tsuchie et al. and Nakamura [15], including:

- “I want to quit working as a nurse.”
- “I want to change hospitals or departments.”
- “I want to continue working as a nurse.”

Responses were recorded on a 5-point Likert scale, with higher scores indicating stronger intention to resign.

### 2.7. Data Collection Procedure

A paid version of Google Forms was used to create the survey and corresponding QR code. After obtaining ethical approval, the research team contacted the director of nursing at each hospital through written and oral explanation to seek consent. Upon receiving permission, the nursing departments were provided with questionnaires (including a QR code) and explanation documents. Ward nurse managers then distributed the materials to eligible preceptors.

Participants who agreed to participate accessed the survey via the QR code on the form. Responses were collected at three separate time points (June, September, December) using the same survey instrument. The first survey was completed via QR code on paper, and for the second and third rounds, the form was distributed via BCC email to avoid disclosing participants’ addresses. Linkage across time points was established through voluntary registration of email addresses, which were deleted after the study’s completion. All responses were anonymous.

### 2.8. Data Analysis

Descriptive statistics (frequencies and percentages) were calculated for demographic data. Participants were categorized into “low” and “high” groups based on their PID:

1. “Not at all,” “Not very much,” and “Neutral” = Low.
2. “Somewhat,” and “Very much” = High.

The mean score of the Preceptor Role Self-Evaluation Scale (mean = 106) was applied to divide participants into high- and low-role-performance groups. Normality was confirmed and two-way ANOVA was conducted with the two factors (perceived difficulty and role performance) for each time point. IBM SPSS Statistics version 26 was used for the analysis.

### 2.9. Ethical Considerations

The study was approved by the Hyogo University Research Ethics Committee (No. 21011). Written explanations of the study purpose and procedures were provided to institutional administrators, and written informed consent was obtained from each participant. The subjects were informed that participation was voluntary and could be withdrawn at any time without penalty. Additionally, they were informed that the data would be utilized solely for this research, analyzed statistically using coded identifiers, and that participants’ privacy would be fully protected. There are no conflicts of interest to declare for this study.

## 3. Results

### 3.1. Participant Characteristics (Table 1)

A total of 76 preceptors participated in the study: six males (7.9%) and 70 females (92.1%). Regarding their work departments, the largest proportion worked in general wards (51 participants, 67.1%), followed by community comprehensive care wards (eight participants, 10.5%). Intensive care units and psychiatric wards had five participants each (6.6%), obstetrics and gynecology had two participants (2.6%), and one participant each worked in pediatric wards, operating rooms, and outpatient departments (1.3%). Two participants (2.6%) belonged to other departments.

### 3.2. Role Performance and Perceived Difficulty in Precepting (Table 2)

In terms of educational background, 39 participants (51.3%) were university graduates, 31 (40.8%) were vocational school graduates, 4 (5.3%) held graduate degrees, and 2 (2.6%) were junior college graduates. Regarding professional qualifications, 71 participants (93.4%) were registered nurses, 3 (3.9%) were public health nurses, and 2 (2.6%) were midwives. For work schedules, 60 participants (78.9%) worked two shifts, 8 (10.5%) worked only day shifts, and 8 (10.5%) worked three shifts.

To examine the relationship between preceptors’ role performance and the perceived difficulty of instructing newly graduated nurses as of June 2022, an independent-samples t-test was conducted between the high- and low-difficulty groups using the subscales of the Preceptor Role Self-Evaluation Scale. The results indicated that the high-difficulty group scored significantly higher than the low-difficulty group on the subscales related to “assessing the condition of the preceptee while aiming to achieve instructional goals and prevent incidents” and “continuing instruction with the support of ward nurses and patients.”

### 3.3. Effects of Perceived Difficulty and Role Performance on Stressors over Time (Table 3)

A two-way ANOVA was conducted to examine the effects of perceived difficulty (high or low) and role performance (high or low) on stressors over three time points (June, September, and December 2022). The subscales of the Nursing Job Stressor Scale were used as dependent variables.

In terms of total strain, a marginally significant trend with the main effect of perceived difficulty was observed in December (*p* = 0.09). Regarding nursing role conflict, the interactions approached significance in June and September (*p* = 0.09, *p* = 0.10). A simple main effects analysis revealed that in the high-difficulty group, participants with high role performance scores had significantly higher nursing role conflict scores than those with low role performance (*p* = 0.05).

For conflicts with physicians, a marginally significant main effect of perceived difficulty was identified in June (*p* = 0.08). In the domain of death and dying, the main effect of perceived difficulty was significant in June (*p* = 0.04), indicating higher stressor scores in the high-difficulty group. Regarding qualitative workload, a significant main effect of role performance was identified in December (*p* = 0.03), suggesting that participants with high role performance perceived less qualitative workload.

The interaction effect on conflict with patients approached significance in December (*p* = 0.10). Simple main effects analysis showed that in the high-difficulty group, participants with high role performance scores had significantly higher conflict with patient scores than those with low role performance (*p* = 0.05).

### 3.4. Effects on Burnout by Perceived Difficulty and Role Performance over Time (Table 4, Figure 1)

Burnout was analyzed based on three dimensions: emotional exhaustion, depersonalization, and personal accomplishment, according to levels of perceived difficulty and role performance over three time points (June, September, and December 2022). For emotional exhaustion, a significant interaction trend was identified at Time 1 (*p* = 0.10). A simple main effects analysis revealed that among participants with low perceived difficulty, those with low role performance had significantly lower emotional exhaustion scores than those with high role performance (*p* = 0.05). No significant effects were identified for depersonalization. Regarding personal accomplishment, significant trend interaction effects were observed at Time 1 (*p* = 0.10) and Time 3 (*p* = 0.04). At both time points, participants in the high-difficulty and high role performance groups reported a significantly lower decline in personal accomplishment than those with low role performance (*p* = 0.10, *p* = 0.04, respectively).

### 3.5. Effects on Turnover Intention over Time (Table 5)

Finally, turnover intention was assessed based on three items: “I want to quit nursing,” “I want to transfer to another hospital or department,” and “I want to continue working as a nurse.” The relationships between these items and perceived difficulty and role performance were examined over three time points.

For the intention to quit nursing, no significant effects were observed at Time 1 or Time 2. However, at Time 3, a significant interaction was identified (*p* = 0.04). In the low-difficulty group, participants with low role performance had significantly lower intention to quit nursing compared to those with high role performance (*p* = 0.04).

For the intention to transfer departments or hospitals, no significant effects were observed at Time 1 or Time 2. However, at Time 3, a significant interaction was identified (*p* = 0.03). Again, in the low-difficulty group, participants with low role performance showed significantly lower intention to transfer than those with high role performance (*p* = 0.04). As for the positive intention to continue working as a nurse, no significant main effects or interactions were observed at any time point.

## 4. Discussion

The study included 76 preceptors from 39 hospitals in Hyogo Prefecture, Japan. Participants were recruited using purposive sampling, and a target sample size of 68 was exceeded, ensuring sufficient statistical power. Most participants were female (92.1%), reflecting the general nursing workforce; however, potential gender differences should be considered. Most of the participants worked in general wards (67.1%), with a few working in other departments. As the workload and stressors may vary by department, the results should be interpreted cautiously when generalizing them to all nursing settings.

This longitudinal study examined preceptors responsible for newly graduated nurses. It explored how their role performance behaviors and PID influenced their mental health. These effects were considered in the context of changes to clinical practicum during the COVID-19 pandemic.

The study is significant in that it elucidates the impact of PID and role performance on stressors, burnout, and intention to resign among nurses who instruct newly graduated nurses.

With the spread of COVID-19, many nursing education institutions suspended or curtailed clinical practicums, opting instead for alternative methods such as online learning and simulations [2,3]. Consequently, newly graduated nurses assigned to clinical settings from 2022 onward were reported to have diminished practical competencies—such as clinical judgment and care skills gained through direct patient interaction—compared to those with conventional practicum experience [7]. This decline in practical skills likely increased the instructional burden on preceptors. In the present study, preceptors exhibited a high level of PID, particularly in tasks such as “understanding the preceptee’s condition while aiming to achieve instructional goals and prevent incidents” and “continuing instruction with the cooperation of ward nurses and patients.” These findings suggest that preceptors were required to engage in instruction involving elevated levels of risk management and situational judgment to compensate for the clinical immaturity of new graduates, resulting in increased instructional complexity and the need for extensive role performance. This structure reflects the burden placed on instructors to compensate for the curtailed practicum experiences caused by the pandemic.

Previous research has shown that such extensive role performance can be a risk factor for burnout and stress [16], and the current findings reinforce concerns that instructional burdens may manifest as psychological strain. In fact, the results indicated that PID and role performance affected several subscales of stressors, including “conflict with physicians,” “conflict with patients,” “nursing role conflict,” “death and dying,” and “qualitative workload.” Notably, those in the high-difficulty and high-performance group tended to score higher on interpersonal stressor subscales, suggesting that preceptors with high responsibility and competence may be more prone to conflicts in workplace relationships and patient interactions [17].

Furthermore, interaction effects between perceived difficulty and role performance were observed in the burnout dimensions of “emotional exhaustion” and “personal accomplishment.” Particularly, participants with both low perceived difficulty and low role performance exhibited lower emotional exhaustion and higher personal accomplishment. This indicates that when both perceived difficulty and role performance are elevated, efforts to fulfill instructional roles may, paradoxically, contribute to greater burnout, as instructors experience the challenges in providing guidance.

Regarding turnover intention, participants with low perceived difficulty and low role performance showed significantly lower levels of negative intentions such as “wanting to quit nursing” or “wanting to transfer to another ward.” This implies that a moderate level of challenge and engagement in instructional activities may positively influence continued occupational motivation [18]. Conversely, positive intentions such as “wanting to continue working as a nurse” were not significantly influenced by perceived difficulty or role performance. This suggests that such positive professional attitudes may be shaped more by individual values and long-term career perspectives than by daily stress or role evaluations [19].

Sánchez-Teruel and Robles-Bello developed the COVID-19 Fear Scale (FCV-19S) and reported that fear of COVID-19 influenced depression and anxiety. Similarly, fear related to COVID-19 may also have affected the stress experienced by preceptors guiding newly graduated nurses [20].

In summary, the findings indicate the impact of instructional difficulty and elevated role performance on the mental health and turnover intention among preceptors responsible for newly graduated nurses who have reduced clinical preparedness due to changes in clinical practicums caused by the COVID-19 pandemic. The educational background of restricted clinical practice during the pandemic has diminished new nurses’ readiness for practice, thereby amplifying the burden on preceptors who strive to compensate for this gap. Going forward, it will be necessary to promote collaboration between basic nursing education and clinical practice settings while also establishing systems that provide psychological support to instructors and promote shared educational responsibilities, such as through team-based instructional models.

However, this study was conducted with nurses from a single prefecture, which may limit the generalizability of our findings. To enhance external validity, future research should include participants from multiple regions to enable more comprehensive and representative data collection. Furthermore, it is important to develop structured programs that provide tailored support not only for newly graduated nurses, but also for the preceptors who mentor them to promote sustainable professional development and effective guidance.

Another notable limitation is the relatively low response rate of 17.8%, which raises the possibility of a response bias. This reduced participation may have been influenced by factors such as survey timing, heavy clinical workload, and survey fatigue. While the demographic characteristics of the respondents were largely consistent with those of the target population, caution should be exercised when interpreting and generalizing the results.

## 5. Conclusions and Practical Implications

This study focused on the psychological impact of educational changes induced by restricted clinical practicums during the COVID-19 pandemic on nurses who instruct newly graduated nurses. It longitudinally examined the effects of PID and role performance on stressors, burnout, and turnover intention. The results revealed that nurses who reported high instructional difficulty and also demonstrated high role performance tended to experience greater stress and burnout, suggesting that the weight of clinical instructional responsibilities manifests as psychological strain. Additionally, turnover intention varied significantly depending on levels of perceived difficulty and role performance.

These findings suggest that the increased effort of clinical instructors to compensate for the diminished practical skills of newly graduated nurses may considerably impact their mental health. Thus, in bridging the gap between post-pandemic nursing education and clinical practice, it is essential to establish systems of psychological and educational support not only for new nurses but also for their preceptors. Specifically, multifaceted interventions such as implementing team-based instructional systems and providing mental health support, including cognitive behavioral therapy, are urgently required.

## Figures and Tables

**Figure 1:**
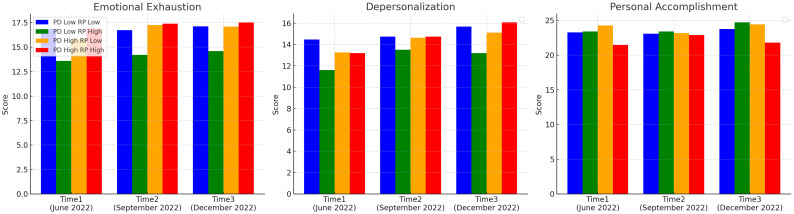
Effects on burnout by perceived difficulty and role performance over time. PD: groups of perceived difficulty; PR: groups of role performance scale.

**Table 1:** Basic attributes of participants.

			*N* = 76
		*n*	%
Gender	Male	6	7.9
	Female	70	92.1
Education	Vocational School	31	40.8
	Junior College	2	2.6
	University	39	51.3
	Postgraduate School	4	5.3
Qualification	Nurse	71	93.4
	Public health nurse	3	3.9
	Midwife	2	2.6
Work Shift	Day Shift Only	8	10.5
	3 Shift Rotation	8	10.5
	2 Shift Rotation	60	78.9
Department	General Ward	51	67.1
	Obstetrics and Gynecology Ward	2	2.6
	Pediatric Ward	1	1.3
	Intensive Care Unit	5	6.6
	Operating Room	1	1.3
	Psychiatric Ward	5	6.6
	Outpatient	1	1.3
	Community Comprehensive Ward	8	10.5
	Other	2	2.6

**Table 2:** Role performance and perceived difficulty in precepting.

	M	SD	M	SD	t	*p*
Providing instruction and evaluation in accordance with the instructional plan.	15.07	3.05	16.19	2.03	−1.91	0.06
Offering explanations of problematic phenomena and psychological support to enable continued work by new nurses.	14.68	2.93	15.55	2.38	−1.41	0.16
Alleviating the new nurse’s tension and compensating for deficiencies.	14.75	3.60	15.74	1.91	−1.57	0.12
Monitoring new nurses’ conditions while aiming to achieve instructional goals and prevent accidents.	14.75	2.35	15.83	2.06	−2.08	0.04
Continuously performing duties other than new nurse instruction.	13.18	3.08	14.15	2.45	−1.51	0.14
Collecting diverse information about new nurses and developing individualized instructional plans reflecting their uniqueness.	14.64	2.34	14.96	1.57	−0.70	0.49
Continuing instruction with cooperation from ward nurses and patients.	13.54	3.47	15.45	2.40	−2.81	0.01
Preceptor role self-evaluation total.	100.61	17.75	109.38	14.21	−2.35	0.02

**Table 3:** Effects of perceived difficulty and role performance on stressors over time.

	Groups of Perceived Difficulty	Low				High												
	Groups of Role Performance Scale	Low		High		Low		High		Main Effect (Groups of Perceived Difficulty)			Main Effect (Groups of Role Performance Scale)			Interaction		
	Time	M	SD	M	SD	M	SD	M	SD	F	*p*	η^2^	F	*p*	η^2^	F	*p*	η^2^
The total strain	Time1 (June 2022)	2.73	0.79	2.58	0.59	2.93	0.54	2.94	0.65	2.67	0.11	0.05	0.17	0.68	0.00	0.23	0.63	0.00
	Time2 (September 2022)	2.85	0.77	2.62	0.31	2.98	0.55	2.73	0.53	0.60	0.44	0.01	2.42	0.13	0.04	0.00	0.98	0.00
	Time3 (December 2022)	2.91	0.65	2.68	0.41	3.11	0.52	2.97	0.45	3.01	0.09	0.05	1.73	0.19	0.03	0.10	0.75	0.00
Nursing role conflict	Time1 (June 2022)	2.79	0.84	2.34	0.86	2.76	0.69	2.99	0.75	2.22	0.14	0.04	0.28	0.60	0.00	2.65	0.09	0.05
	Time2 (September 2022)	2.91	0.78	2.12	0.61	2.95	0.66	2.71	0.71	2.83	0.10	0.05	7.58	0.01	0.12	2.14	0.10	0.04
	Time3 (December 2022)	2.79	0.71	2.46	0.60	2.96	0.51	2.92	0.68	3.56	0.06	0.06	1.19	0.28	0.02	0.75	0.39	0.01
Conflict with physicians	Time1 (June 2022)	2.59	1.03	2.48	0.73	3.08	0.85	2.85	1.02	3.10	0.08	0.05	0.47	0.50	0.01	0.06	0.80	0.00
	Time2 (September 2022)	2.44	1.12	2.38	0.62	2.73	0.89	2.39	0.80	0.37	0.54	0.01	0.69	0.41	0.01	0.34	0.56	0.01
	Time3 (December 2022)	2.71	0.96	2.32	0.58	2.99	0.70	2.76	0.75	3.07	0.09	0.05	2.23	0.14	0.04	0.14	0.70	0.00
Death and dying	Time1 (June 2022)	2.15	1.02	1.85	0.98	2.42	0.92	2.58	0.71	4.26	0.04	0.07	0.08	0.78	0.00	0.90	0.35	0.02
	Time2 (September 2022)	2.47	1.02	2.00	0.84	2.62	0.88	2.30	0.87	0.86	0.36	0.02	2.60	0.11	0.05	0.09	0.76	0.00
	Time3 (December 2022)	2.43	0.92	2.08	0.75	2.61	0.95	2.50	0.95	1.50	0.23	0.03	0.90	0.35	0.02	0.27	0.61	0.00
Qualitative workload	Time1 (June 2022)	2.92	0.90	2.42	0.89	2.95	0.77	2.93	0.58	1.67	0.20	0.03	1.51	0.22	0.03	1.35	0.25	0.02
	Time2 (September 2022)	3.01	0.94	2.76	0.69	3.18	0.55	2.91	0.65	0.67	0.42	0.01	1.91	0.17	0.03	0.00	0.96	0.00
	Time3 (December 2022)	3.23	0.76	2.74	0.78	3.22	0.58	2.95	0.46	0.34	0.56	0.01	4.93	0.03	0.08	0.38	0.54	0.01
Quantitative workload	Time1 (June 2022)	3.15	0.91	3.34	0.48	3.33	0.62	3.25	0.73	0.06	0.81	0.00	0.10	0.75	0.00	0.48	0.49	0.01
	Time2 (September 2022)	3.36	0.77	3.34	0.45	3.27	0.82	3.03	0.83	0.97	0.33	0.02	0.43	0.51	0.01	0.31	0.58	0.01
	Time3 (December 2022)	3.36	0.73	3.34	0.73	3.44	0.59	3.44	0.56	0.28	0.60	0.01	0.00	0.95	0.00	0.00	0.96	0.00
Conflict with patients	Time1 (June 2022)	2.67	0.84	2.85	0.58	3.03	0.77	2.77	1.02	0.39	0.54	0.01	0.03	0.86	0.00	0.99	0.32	0.02
	Time2 (September 2022)	3.30	0.73	2.60	0.81	3.13	0.96	2.70	0.98	0.02	0.89	0.00	5.71	0.02	0.09	0.32	0.57	0.01
	Time3 (December 2022)	2.93	1.05	3.00	0.88	3.34	0.60	2.77	0.78	0.16	0.69	0.00	1.33	0.25	0.02	2.12	0.10	0.04
Conflict with other nursing staff	Time1 (June 2022)	2.71	0.95	2.71	0.94	2.91	0.74	3.01	0.76	1.16	0.29	0.02	0.05	0.83	0.00	0.05	0.83	0.00
	Time2 (September 2022)	2.73	0.95	2.89	0.40	2.99	0.63	2.92	0.68	0.62	0.44	0.01	0.05	0.83	0.00	0.34	0.56	0.01
	Time3 (December 2022)	2.85	0.81	2.83	0.48	3.19	0.63	3.14	0.42	3.96	0.05	0.07	0.04	0.85	0.00	0.01	0.94	0.00

F = F statistic; *p* = probability value; η^2^ = effect size (eta squared).

**Table 4:** Effects on burnout by perceived difficulty and role performance over time.

	Groups of Perceived Difficulty	Low				High												
	Groups of Role Performance Scale	Low		High		Low		High		Main Effect (Groups of Perceived Difficulty)			Main Effect (Groups of Role Performance Scale)			Interaction		
	Time	M	SD	M	SD	M	SD	M	SD	F	*p*	η^2^	F	*p*	η^2^	F	*p*	η^2^
Emotional exhaustion	Time1 (June 2022)	16.87	4.29	13.60	4.40	15.84	6.15	16.93	3.73	0.78	0.38	0.01	0.70	0.41	0.01	2.79	0.10	0.05
	Time2 (September 2022)	16.73	4.82	14.20	4.08	17.26	5.84	17.40	4.37	1.97	0.17	0.03	0.81	0.37	0.01	1.01	0.32	0.02
	Time3 (December 2022)	17.13	5.07	14.60	4.09	17.11	6.27	17.53	4.82	1.05	0.31	0.02	0.55	0.46	0.01	1.09	0.30	0.02
Depersonalization	Time1 (June 2022)	14.47	5.69	11.60	3.86	13.26	6.11	13.20	5.35	0.02	0.89	0.00	0.99	0.32	0.02	0.91	0.34	0.02
	Time2 (September 2022)	14.73	5.34	13.50	6.31	14.63	5.82	14.73	5.05	0.14	0.71	0.00	0.14	0.71	0.00	0.20	0.66	0.00
	Time3 (December 2022)	15.67	5.22	13.20	6.14	15.11	6.02	16.07	4.80	0.60	0.44	0.01	0.26	0.61	0.00	1.33	0.25	0.02
Personal accomplishment	Time1 (June 2022)	23.27	3.56	23.40	3.66	24.26	2.77	21.47	3.18	0.29	0.59	0.01	2.37	0.13	0.04	2.86	0.10	0.05
	Time2 (September 2022)	23.07	4.22	23.40	3.81	23.16	3.48	22.87	2.29	0.06	0.81	0.00	0.00	0.98	0.00	0.11	0.74	0.00
	Time3 (December 2022)	23.73	2.81	24.70	3.27	24.42	2.43	21.80	4.09	1.72	0.20	0.03	0.96	0.33	0.02	4.52	0.04	0.08

F = F statistic; *p* = probability value; η^2^ = effect size (eta squared).

**Table 5:** Effects on turnover intention over time.

	Groups of Perceived Difficulty	Low				High												
	Groups of Role Performance Scale	Low		High		Low		High		Main Effect (Groups of Perceived Difficulty)			Main Effect (Groups of Role Performance Scale)			Interaction		
	Time	M	SD	M	SD	M	SD	M	SD	F	*p*	η^2^	F	*p*	η^2^	F	*p*	η^2^
Wants to quit working as a nurse	Time1 (June 2022)	3.20	1.42	2.40	1.35	2.63	1.38	2.73	1.16	0.11	0.74	0.00	0.96	0.33	0.02	1.59	0.21	0.03
	Time2 (September 2022)	3.13	1.30	2.30	1.57	3.11	1.52	3.00	1.13	0.82	0.37	0.01	1.60	0.21	0.03	0.97	0.33	0.02
	Time3 (December 2022)	3.07	1.22	1.90	1.37	2.95	1.61	3.33	1.23	3.13	0.08	0.05	1.10	0.30	0.02	4.37	0.04	0.07
Wants to switch hospitals	Time1 (June 2022)	2.80	1.42	2.50	1.08	2.68	1.42	3.13	1.25	0.53	0.47	0.01	0.04	0.83	0.00	1.12	0.30	0.02
	Time2 (September 2022)	3.07	1.39	2.00	1.05	3.11	1.33	3.00	1.31	2.24	0.14	0.04	2.85	0.10	0.05	1.92	0.17	0.03
	Time3 (December 2022)	3.33	1.18	2.40	1.35	2.95	1.47	3.60	1.24	1.32	0.25	0.02	0.16	0.69	0.00	5.02	0.03	0.08
Wants to continue working as nurse	Time1 (June 2022)	3.13	1.30	3.90	1.20	3.63	1.21	3.67	1.11	0.17	0.68	0.00	1.54	0.22	0.03	1.28	0.26	0.02
	Time2 (September 2022)	3.27	1.39	3.00	1.83	3.47	1.35	3.33	1.23	0.51	0.48	0.01	0.29	0.59	0.01	0.03	0.87	0.00
	Time3 (December 2022)	3.07	1.49	3.60	1.26	3.47	1.35	3.20	1.32	0.00	0.99	0.00	0.13	0.72	0.00	1.22	0.27	0.02

F = F statistic; *p* = probability value; η^2^ = effect size (eta squared).

## Data Availability

The de-identified data underlying the results presented in this study are available upon request to the corresponding author.

## Abbreviations

The following abbreviations are used in this manuscript:

COVID-19	Coronavirus Disease
WHO	World Health Organization
PHEIC	Public Health Emergency of International Concern
NJSS	Nursing Job Stressor Scale
MBI	Maslach Burnout Inventory
